# Total synthesis: an enabling science

**DOI:** 10.3762/bjoc.19.36

**Published:** 2023-04-19

**Authors:** Bastien Nay

**Affiliations:** 1 Laboratoire de Synthèse Organique, École Polytechnique, CNRS, ENSTA, Institut Polytechnique de Paris, 91128 Palaiseau, France

**Keywords:** chemical complexity, natural products, synthetic methodologies, total synthesis

Total synthesis is a topic strongly related to synthetic methodology developments and to natural product isolation or biosynthesis. Thus, thematic issues dealing with total synthesis in the *Beilstein Journal of Organic Chemistry* have naturally been published in these fields, such as "Transition-metal and organocatalysis in natural product synthesis" (edited by D. Chen and D. Ma) [1] or "Natural products in synthesis and biosynthesis" (edited twice by J. Dickschat) [2–3]. Previously, the early thematic issue "Indolizidines and quinolizidines: natural products and beyond" (edited by J. P. Michael) from 2007 [4] displayed some of the very first total syntheses ever published in the *Beilstein Journal of Organic Chemistry*, such as those of frog indolizidine alkaloids [5–6]. However, no thematic issue specifically related to total synthesis has been published before, and we thought it would be time to fill this gap. Although innovative developments are constantly needed to improve methods and strategy, keeping in mind the difficulty of performing transformations on highly functionalized compounds, the total synthesis of complex natural products is indeed mature in terms of efficiency, practicality, economy, and scalability. In short, it has become an enabling science.

Total synthesis is the science of constructing molecules from simple starting materials. It often deals with complex molecular architectures that require a thorough retrosynthetic problem-solving analysis [7]. Total synthesis and the discovery of new synthetic methodologies have always been intimately related. Specific methodologies have often been developed to synthesize valuable compounds, while total synthesis has often been a pretext to demonstrate the value of a new synthetic reaction [8–9]. Old and recent achievements show that connections to other disciplines are important to the success of total synthesis and can be a source of new discoveries. Indeed, as a science allowing the preparation of useful functional compounds, it is strongly connected to biological and medicinal studies, while the development of natural-product-based tools for chemical biology often requires the construction of complex molecules [10–11]. Incidentally, the term "total synthesis" is not limited to natural products but also, sometimes, targets complex drugs [12–13]. Furthermore, it is possible to test biosynthetic hypotheses concerning natural products through synthetic approaches (biomimetic synthesis) [14]. In addition, total syntheses have also been achieved with enzymes, strengthening the links to biology [15].

Total synthesis is not limited to academic laboratories but rather also pursued in industry, where a particular efficiency and economy of tasks is of paramount importance [12], as illustrated in this thematic issue with the synthesis of pheromones [16]. This requires permanent technological progress. Thus, the recent boom of artificial intelligence, machine learning, and computational chemistry for retrosynthetic analyses and beyond foreshadows a renewed interest in harvesting increasingly complex synthetic strategies for industrial processes. Although it can be useful in operating routine processes, AI will not replace human creativity [17]. In terms of discoveries in organic chemistry, total synthesis is a fruitful feed, and serendipity has well been exploited. Even dead ends, yet always heartbreaking for synthetic chemists, still provide a wealth of useful information for the chemical community [18]. Finally, this is not to forget that organic chemistry, which is above all an experimental science, is performed daily by researchers in the laboratory, and some of these lives can be truly inspiring to current and future generations.

**Figure 1:**
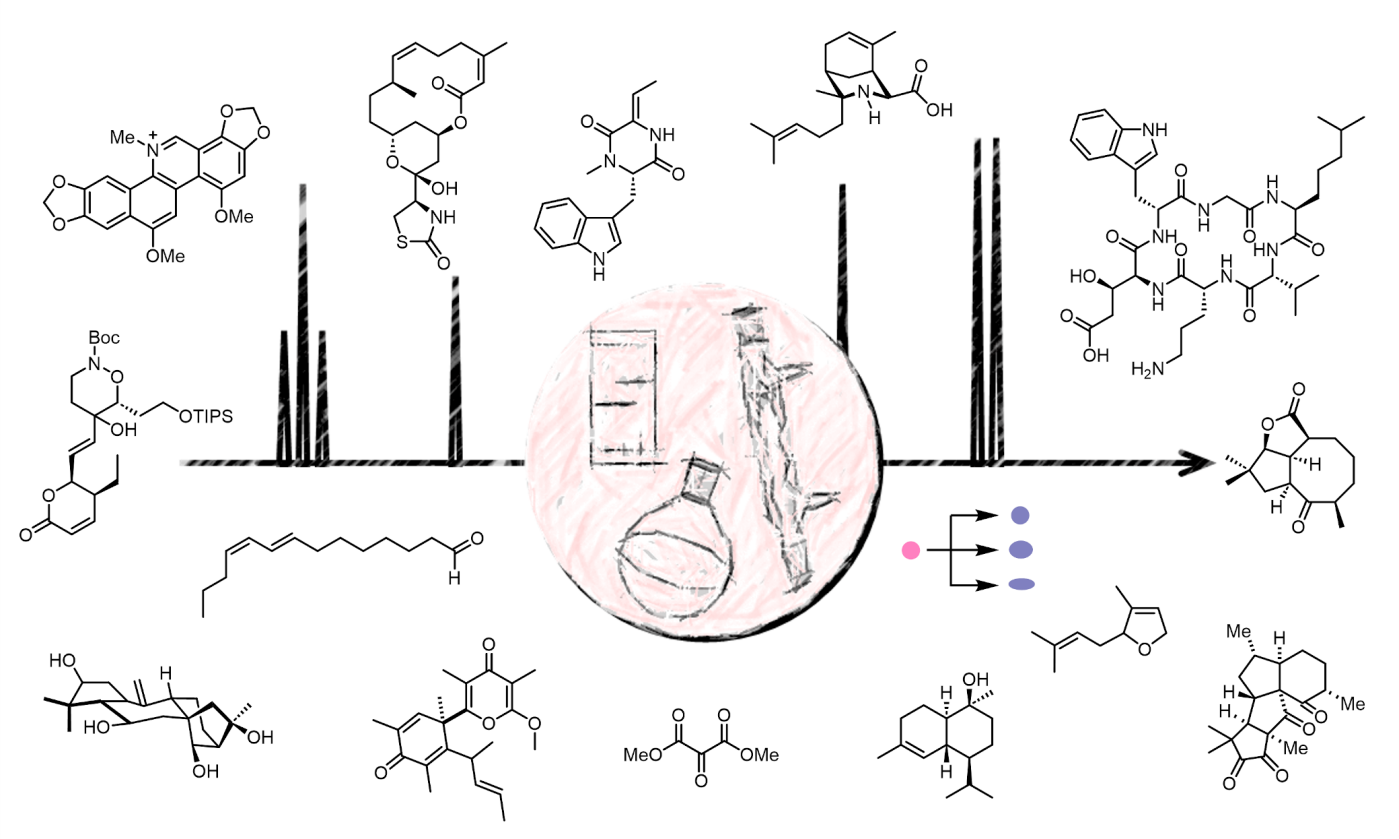
A summary of compounds targeted in this thematic issue.

It was the aim of this thematic issue to cover any of the previous remarks. Thanks to the contributions of talented and enthusiastic authors, we were able to gather articles on total synthesis (Figure 1), not only illustrating the utility but also the vitality of this field.

I warmly thank all the authors of this thematic issue for their beautiful contributions. I also thank the Editorial Team of the *Beilstein Journal of Organic Chemistry* for their significant efforts in checking, formatting, and finally subtilizing all these articles.

Bastien Nay

Palaiseau, April 2023
